# Clonify: unseeded antibody lineage assignment from next-generation sequencing data

**DOI:** 10.1038/srep23901

**Published:** 2016-04-22

**Authors:** Bryan Briney, Khoa Le, Jiang Zhu, Dennis R. Burton

**Affiliations:** 1 Department of Immunology and Microbial Science, The Scripps Research Institute, La Jolla, CA 92037, USA; 2 International AIDS Vaccine Initiative Neutralizing Antibody Center, The Scripps Research Institute, La Jolla, CA 92037, USA; 3 Center for HIV/AIDS Vaccine Immunology and Immunogen Discovery, The Scripps Research Institute, La Jolla, CA 92037, USA; 4 Ragon Institute of Massachusetts General Hospital, Massachusetts Institute of Technology, and Harvard University, Boston, MA 02142, USA

## Abstract

Defining the dynamics and maturation processes of antibody clonal lineages is crucial to understanding the humoral response to infection and immunization. Although individual antibody lineages have been previously analyzed in isolation, these studies provide only a narrow view of the total antibody response. Comprehensive study of antibody lineages has been limited by the lack of an accurate clonal lineage assignment algorithm capable of operating on next-generation sequencing datasets. To address this shortcoming, we developed Clonify, which is able to perform unseeded lineage assignment on very large sets of antibody sequences. Application of Clonify to IgG+ memory repertoires from healthy individuals revealed a surprising lack of influence of large extended lineages on the overall repertoire composition, indicating that this composition is driven less by the order and frequency of pathogen encounters than previously thought. Clonify is freely available at www.github.com/briney/clonify-python.

Upon initial antigen encounter, a naïve B cell proliferates and accumulates somatic mutations in the antibody genes that strengthen the antibody-antigen interaction. The result is a family of clonally related B cells encoding similar but unique antibody genes. An antibody clonal lineage is then defined as the population of antibodies encoded by B cells that originate from a single naive B cell. Although genetically similar, individual members of an antibody clonal lineage often display functional differences that are important for a robust antibody response to a pathogen^1^.

Next-generation sequencing (NGS) platforms have dramatically increased our ability to perform detailed analysis of antibody repertoires and responses to immunization and infection^2^,^3^,^4^,^5^. While these platforms have enabled vastly greater sequencing depth, their throughput still pales in comparison to the size of the total antibody repertoire. Therefore, although cross-sample comparisons may indicate great individual sequence variability, this may stem from insufficient sampling depth rather than from biologically relevant variation. Indeed, higher-level analyses show remarkable repertoire consistency between individuals and over time^6^,^7^,^8^, but the dynamics of individual antigen-specific lineages are likely to be masked when analyzing from such a high level. In our view, the clonal lineage is likely the ‘sweet spot’ for analyzing and understanding antibody responses to immunization and infection, but, due to the lack of unseeded (see below) antibody clonal lineage assignment methods, these studies have not been possible.

When assessing the clonal relatedness of antibody sequences from NGS data, there are two types of antibody lineage assignment. The first type, ‘seeded’ lineage assignment, involves identifying sequences from a dataset that belong to the same clonal lineage as one or more known (or ‘seed’) antibody sequences. In the second assignment class, termed ‘unseeded’ lineage assignment, the clonal relationships of all sequences in a dataset are identified. Various forms of seeded lineage assignment have been previously used to study the maturation of individual lineages^1^,^9^,^10^,^11^,^12^,^13^,^14^. Owing to the difficulty of unseeded lineage assignment, however, there have been no large-scale studies of antibody repertoire clonality beyond individual lineages. Unseeded lineage assignment is much more computationally demanding than seeded lineage assignment, requiring pairwise comparisons between each sequence in the dataset with all other sequences in the dataset. Additionally, assignment accuracy becomes much more important when considering unseeded lineage assignment. In seeded assignment, it is possible (and often, advantageous) to use an overly inclusive algorithm since the single resulting lineage can be manually curated following assignment. In contrast, unseeded assignment would typically involve many thousands of lineages, making it unfeasible to manually correct the assignments in each lineage. Therefore, an unseeded lineage assignment algorithm must be highly accurate as well as computationally efficient.

Sequencing errors are a very important consideration for the analysis of antibody NGS data. Since errors are essentially randomly distributed, it is extremely difficult to distinguish legitimate somatic mutations from apparent substitutions that are the result of sequencing error. The most commonly used error correction method is to cluster sequences using a homology threshold and build a single consensus sequence for each cluster^1^,^11^,^12^. While this technique can effectively eliminate many sequencing errors, closely related antibodies will often be assigned to the same cluster, and legitimate antibody diversity will be lost. Other studies use single-molecule barcoding to uniquely label viral or antibody transcripts, sequence at high coverage and build consensus sequences from multiple reads of the same transcript^15^,^16^,^17^. An important criticism of such studies, however, involves their use of insufficient barcode diversity to produce truly unique transcript barcoding^18^.

We have developed a modified cDNA barcoding strategy with sufficient diversity to ensure truly unique transcript barcoding and have created an unseeded clonal lineage assignment algorithm capable of highly accurate antibody lineage assignment using large NGS datasets. With these tools we were able to perform, for the first time, a detailed analysis of clonality in the human IgG+ memory repertoire. The tools provide a framework for future analyses of antibody responses to infection and immunization.

## Results

### Error and bias correction with unique antibody identifiers

Since, by definition, the naïve B cell subset does not contain any clonally related sequences, we focused our lineage analysis on the IgG+ memory B cell subset. We isolated IgG+ memory B cells from 8 healthy human donors and sequenced the encoded antibody heavy chains (Supplementary Table 1). To minimize sequencing errors and amplification bias, we adapted a previously used barcoding strategy^17^ that involves labeling transcripts with unique random sequence tags. Based on the estimated number of input B cells (15,000–50,000 per sample), we selected a random tag length of 20 nucleotides, which theoretically produces 4^20^ (roughly one trillion) unique antibody identifiers (UAIDs) and provides a very high likelihood (97.2%) that each antibody transcript is uniquely labeled.

**Figure 1:**
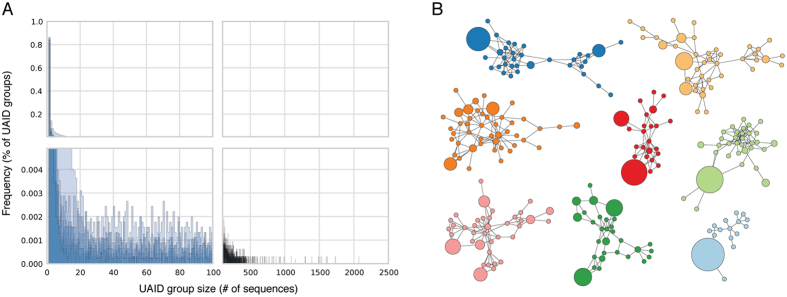
Error and bias correction using unique antibody identifiers (UAIDs). (**A**) Separately for each donor, raw antibody sequences were binned by UAID and the size of each UAID bin was determined. Shown is a histogram of bin sizes, with each donor represented by a single, semi-transparent plot. (**B**) Force-directed network plots of ‘lineages’ built from raw sequences drawn from a single UAID bin. Each plot represents a single UAID bin, and one UAID bin per donor is shown. As the sequences in each network plot were taken from a single UAID bin, they represent multiple reads of the same RNA transcript. Therefore, the sequence diversity in each of the plots is due entirely to sequencing and amplification error.

To investigate the degree to which amplification biases affect the sequenced antibody repertoire composition, we grouped sequences by UAID and determined the size of each UAID group (Fig. 1A). We discovered several UAID groups containing over 1000 sequences, indicating that amplification bias had skewed the representation of these transcripts by multiple orders of magnitude. Compounding the problem, sequencing errors are able to convincingly mimic the natural antibody maturation process^9^. To examine the effect of a lack of error correction on the generation of accurate clonal lineages, lineage assignments were made using raw sequences without UAID correction and a single lineage was selected from each donor (Fig. 1B). If UAID correction was now carried out, each of the lineages in Fig. 1B was found to originate from a single antibody transcript. In other words, all of the diversity contained in these lineages is due to sequencing error, not the antibody maturation process; sequencing errors and disproportionate amplification have combined to produce artifactual ‘lineages’ that contain no information about naturally occurring antibody diversification.

### Clonal lineage assignment

**Figure 2:**
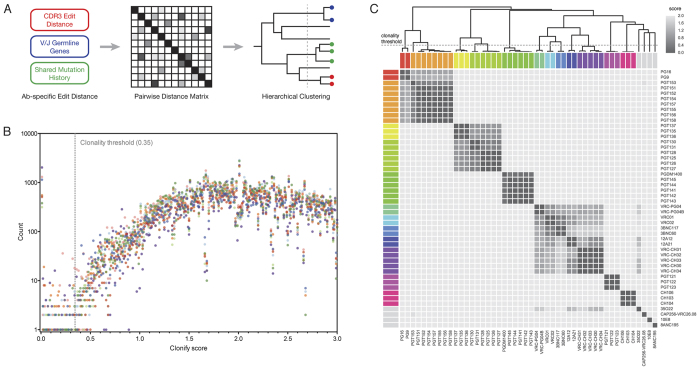
The Clonify algorithm for antibody lineage assignment. (**A**) Schematic of the Clonify algorithm, which consists of two major parts: the calculation of an antibody-specific edit distance for each sequence pair and the assembly of these scores into a pairwise distance matrix, followed by hierarchical clustering of the antibody sequences. (**B**) Separately for each of the eight donors, 1000 sequences were randomly selected and the pairwise distance was calculated for each sequence pair. Performing all-versus-all comparisons on 1000 sequences results in the computation of 499,500 pairwise distance scores. The frequency of each distance score is shown (the X-axis is truncated at 3.0 for clarity). The trough in score frequencies, which was used to assign the clustering threshold, is indicated. (**C**) Clonify was used to group a panel of HIV broadly neutralizing antibody (bnAb) sequences into clonal lineages. The pairwise distances computed by Clonify were used to create a distance matrix, with dark grey indicating high similarity (low distance score). Known clonal lineages are indicated by color on the top and left sides of the distance matrix and known singletons (sequences without any clonal relatives in the bnAb panel) are all colored light grey. Sequences were clustered by Clonify score, and the resulting dendrogram is shown above the distance matrix. The clonality threshold is indicated with a dashed line across the clustering dendrogram.

To permit unseeded lineage analysis of corrected antibody sequences, we developed the Clonify algorithm, which is shown schematically in Fig. 2A. Briefly, a distance matrix is calculated using an antibody-specific distance metric for each pair of sequences and the sequences are hierarchically clustered into lineages. To determine an appropriate clustering threshold, we used Clonify to calculate distance scores for 1000 UAID-corrected sequences from each of the 8 donors. Scores were binned and the frequency of each bin was calculated. There was a distinct divide in the scoring frequencies, indicating clear separation between the scores of related and unrelated sequences (Fig. 2B). We next sought to test the accuracy of Clonify’s lineage assignments. Unfortunately, the sort of large, annotated datasets of clonal lineages that would allow robust accuracy assessment are not available. Instead, we assayed the accuracy of the Clonify algorithm using three-pronged resources: a relatively small dataset of known clonally-related antibody sequences, larger datasets of presumably clonally-related antibody sequences that were identified using a seeded lineage assignment algorithm^19^, and several large NGS datasets from normal human donors for which antibody clonal relationships are unknown.

We first assembled a panel of HIV broadly neutralizing antibody (bnAb) sequences that contains multiple groups of known clonally related sequences^10^,^20^,^21^,^22^,^23^,^24^,^25^,^26^,^27^,^28^. Overwhelmingly, Clonify correctly grouped sequences into lineages (Fig. 2C) and appropriately segregated singletons (sequences without known clonal relatives in the bnAb dataset). Notably, although the bnAb dataset contains several genetically similar ‘VRC01-class’ lineages^10^,^20^,^21^ from multiple donors, Clonify correctly assigns these lineages. Further, the two cases in which Clonify made putatively incorrect assignments, that is excluding PGT153 from the PGT151 lineage^26^ and assigning PGT130/131 and PGT125-128 to separate lineages^23^ are the lineages for which evidence of a clonal relationship is weakest. In the case of the PGT150 lineage, PGT153 has very low HCDR3 homology to other PGT150 lineage members (39–46%; Figure S2) and shares very few somatic mutations with other PGT151 members^26^. In fact, the variable region of PGT153 is so distinct that it is assigned a different D_H_ gene and V_H_ allele to the rest of the PGT150 family by both IMGT and IgBLAST (Table S3). PGT130/131 and PGT125–128 are a similar case, with substantial divergence in the HCDR3 and minimal shared somatic mutation^23^. If these sequences are true somatic relatives, they appear to have diverged from the rest of the lineage very early and matured independently.

We next compared Clonify to a previously published seeded lineage assignment algorithm^19^. Using two data sets, in which sequences were identified by the seeded assignment algorithm as clonally related to the HIV bnAbs PGT141 or PGV04, Clonify was run on putative PGT141- or PGV04-like sequences. For both datasets, we found that Clonify closely reproduced the results of the seeded lineage assignment algorithm. In the case of the PGT141 lineage, 274 putative PGT141-like sequences were identified by the seeded algorithm and 259 of those sequences (94.5%) were assigned to a single lineage by Clonify. Lineage sizes and representative junctions for each Clonify-assigned lineage are shown in Table S4. Similar results were seen for the PGV04 lineage. Of 4267 putative PGV04-like sequences identified by the seeded algorithm, 4002 were assigned to a single lineage by Clonify (93.8%; Table S5).

**Figure 3:**
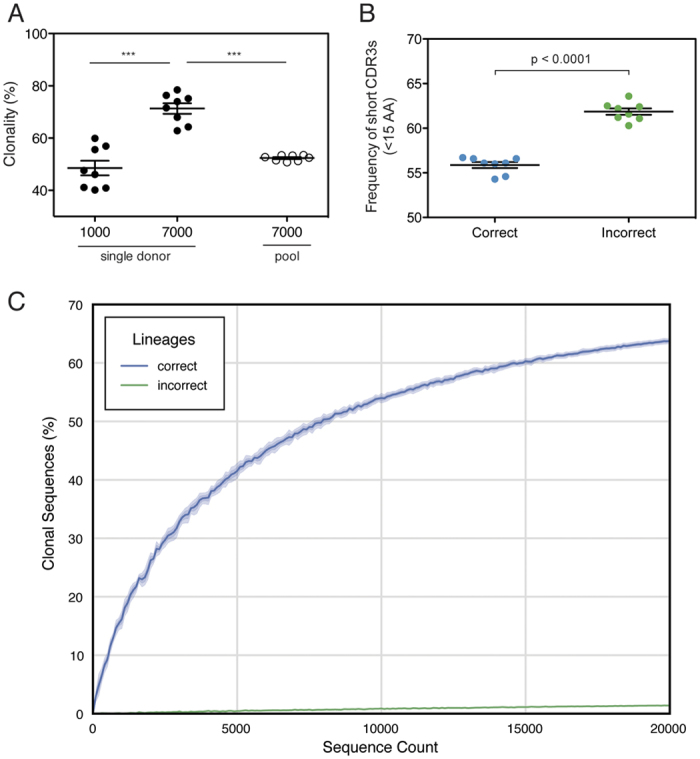
Accuracy of the Clonify algorithm. (**A**) For each donor, either 1000 or 7000 sequences were randomly selected and assigned to lineages. The clonality of each sample was determined, which represents the frequency of sequences belonging to a lineage with at least two members. Eight sequence pools were then constructed, containing 1000 randomly selected sequences from seven of the eight donors such that each donor was left out of a single pool. Lineages were assigned and the level of clonality was determined for each pool. The mean clonality for the multi-donor sequence pools was statistically indistinguishable from the single donor sequence sets containing 1000 sequences. Clonality of single donor sequence sets containing 7000 sequences was found to be significantly higher than both the single donor sequence sets with 1000 sequences (P < 0.0001) and the multi-donor pools (p < 0.0001) by two-tailed Student’s T-test. (**B,C**) Multi-donor sequence pools of increasing size were created by randomly selecting an equal number of sequences from each donor. Lineages were iteratively assigned for each multi-donor pool, and the frequency of ‘incorrect’ assignments, which we define as sequences assigned to a lineage containing primarily sequences from a different donor, was calculated. The frequency of ‘correct’ and ‘incorrect’ assignments is shown in (**C**). The frequency of short HCDR3 regions (<15 amino acids) was calculated for the ‘correct’ and ‘incorrectly’ assigned sequences for each donor (**B**). Incorrectly assigned sequences encoded significantly shorter HCDR3s than did correctly assigned sequences (p < 0.0001, two-tailed Student’s T-test).

Since clonal lineages, by their most literal definition, must originate from a single naive B cell, true lineages should not be shared between individuals. It follows, then, that an accurate lineage assignment algorithm, when given a pool of sequences from multiple donors, should build clonal lineages consisting of sequences exclusively from a single donor. We randomly selected two datasets, containing either 1000 or 7000 sequences, from each of our donors, assigned the sequences from each dataset to clonal lineages, and determined the frequency of sequences that belonged to a lineage with at least two members. As expected, due to the deeper sampling in the 7000 sequence datasets, the level of clonality was significantly higher in the larger dataset in each instance (Fig. 3A). We then made 8 leave-one-out cross-validation (LOOCV) sequence pools containing 1000 sequences from 7 of the 8 donors such that each of the 8 donors was left out of one of the sequence pools. Analysis of the LOOCV sequence pools provides a simple test for lineage assignment accuracy: if, at one extreme, the lineage assignment algorithm makes no distinction between sequences from multiple donors, the increased depth provided in the LOOCV pool should result in a frequency of clonally-related sequences equivalent to the single-donor pool of 7000 sequences. At the opposite extreme, if assigned lineages exclusively contain sequences from a single donor, the frequency of clonally related sequences in the LOOCV pool will be equivalent to the frequency seen in the single-donor datasets containing 1000 sequences. As shown in Fig. 3A, the frequency of clonally related sequences in the LOOCV pools is statistically indistinguishable from the single-donor pools containing just 1000 sequences, indicating a high level of algorithmic distinction between sequences from different donors.

To more precisely calculate the frequency of assignments to lineages containing sequences from multiple donors, we iteratively selected increasing numbers of sequences from each of the single-donor sequence sets. The sequences were pooled into multi-donor sequence sets, lineage assignments were made, and the frequencies of ‘correct’ assignments (sequences belonging to lineages containing sequences from a single donor) and ‘incorrect’ assignments were calculated (Fig. 3C). Even using this overly strict definition of clonality, the vast majority of sequences (>97%) are ‘correctly’ assigned. Indeed, among incorrectly assigned sequences, we found a HCDR3 length distribution that skewed toward short HCDR3s (Fig. 3B). Since there is less junctional diversity among sequences with short HCDR3s, the likelihood of multiple donors coincidentally expressing very similar antibodies with short HCDR3s is much higher. Therefore, even though a strict definition of clonal lineages (one in which cross-donor lineages are impossible) would suggest that all lineages containing sequences from multiple donors are the result of incorrect assignment, the low diversity of the short HCDR3 population results in a higher frequency of inter-donor sequences that are genetically indistinguishable from clonally related sequences.

### Comparison between Clonify and other unseeded lineage assignment algorithms

To gauge the accuracy of Clonify relative to other unseeded lineage assignment algorithms, we evaluated the performance of each algorithm in two ways. The first, which determines the accuracy of lineage assignment on highly mutated HIV-1 bnAbs of known clonal relationships, is designed to test the inclusiveness of each algorithm. The second test compares the stringency of each algorithm using a pool of antibody sequences isolated from eight healthy donors. Five algorithms were selected for comparison and are referred to by the senior author and year of publication: Quake2013a^29^, Quake2013b^16^, Boyd2014^30^, Church2014^8^, and Martinez-Bernetche2015^31^. Details of each algorithm can be found in the Supplementary Methods.

**Figure 4:**
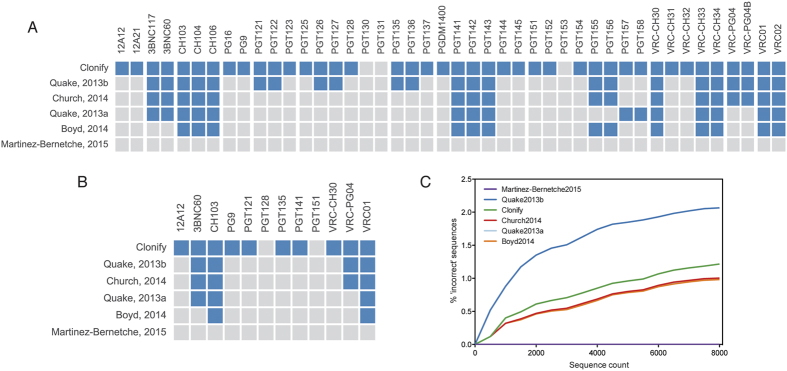
Comparison of unseeded lineage assignment algorithms. (**A**) HIV bnAb sequences were assigned to lineages with each of 6 algorithms. Antibody sequences that were correctly assigned are indicated with blue squares, incorrectly assigned sequences are gray. (**B**) For each algorithm, correctly assigned lineages (lineages for which every antibody is correctly assigned) are indicated in blue, incorrect lineages are indicated in gray. (**C**) For each unseeded lineage assignment algorithm, the frequency of sequences assigned to lineages containing sequences from multiple donors (‘incorrect’ sequences) is shown.

To compare algorithmic inclusiveness, we used the same panel of HIV bnAb sequences shown in Fig. 2C, except that all singleton antibody sequences (those belonging to a lineage with only a single member) were removed. These sequences were assigned to lineages by each algorithm, and the number of correctly assigned antibody sequences was determined (Fig. 4A). Clonify performed the best, correctly assigning 41 of 44 antibodies (93%). Quake2013b assigned 24 antibody sequences correctly (55%). Church2014, Quake2013b and Boyd2014 each assigned approximately one third of the sequences correctly (39%, 34% and 30%, respectively). Martinez-Bernetche2015 performed the least well, assigning each antibody to a separate lineage. We next measured assignment accuracy at the level of the lineage. For a lineage to be counted as correct, every member of the lineage must be correctly assigned. This is a distinct measurement from that shown in Fig. 4A, because at the antibody level, a partially correct lineage earns partial credit; when measuring accuracy at the lineage level, completely correct lineages are required. Of the 12 lineages, Clonify assigned 10 of them completely correctly (83%; Fig. 4B). Interestingly, although Quake2013b performed better than Church2014 when looking at individual antibody sequences, Quake2013b and Church2014 performed identically at the lineage level, with each assigning 4 lineages completely correctly (29%), Quake2013a correctly assigned 3 lineages (21%), Boyd2014 correctly assigned 2 lineages (14%), and Martinez-Bernetche did not assign any lineages completely correctly. It is important to note that the HIV bnAb inclusiveness test is extremely difficult and, in many cases, is a scenario for which these previously published algorithms were not designed. We provide these results not to diminish the usefulness of these algorithms, which have previously been shown to be highly accurate on data sets for which they were designed, but only to demonstrate the difficulty of performing unseeded lineage assignment when lineages contain highly divergent antibody sequences.

Our second comparison uses a pool of sequences derived from eight healthy donors. As in Fig. 3C, lineages are assigned from the cross-donor pool, and the fraction of incorrectly assigned sequences (sequences assigned to lineages containing sequences from multiple donors) is computed. Strikingly, although Clonify is inclusive enough to correctly assign nearly all of the HIV bnAbs, it assigns cross-donor lineages at approximately the same rate as the much less inclusive algorithms Church2014, Quake2013a and Boyd2014 (Fig. 4C). The second-highest scoring algorithm on the inclusiveness test (Quake2013b) is the least discriminating algorithm by a large margin: over 2% of sequences in the 8,000 sequence cross-donor pool are assigned to lineages containing sequences from multiple donors, compared to approximately 1% for Clonify. These results definitively show that Clonify is more inclusive than other unseeded lineage assignment algorithms and, critically, accomplishes this inclusiveness while retaining high stringency.

### Antibody repertoire clonality

**Figure 5:**
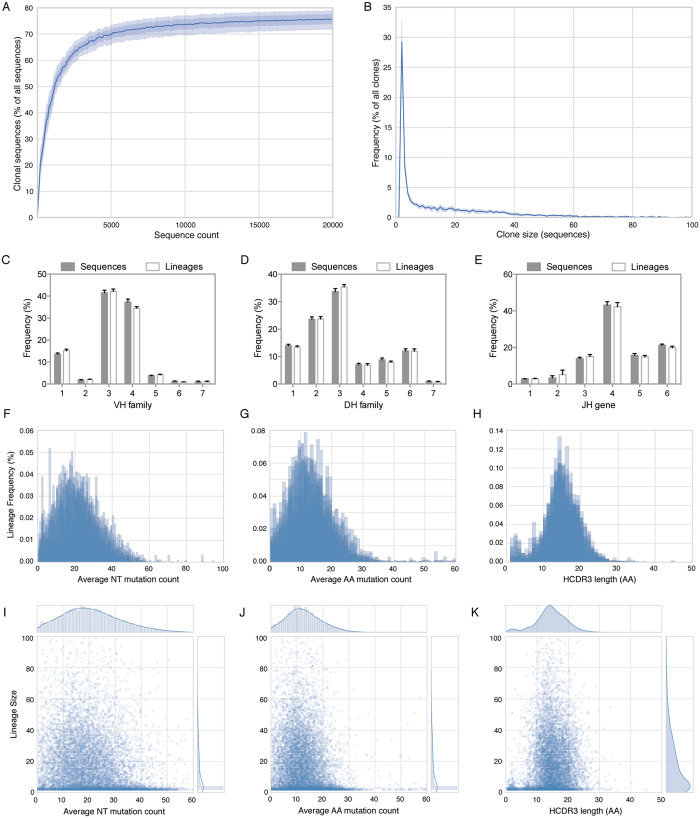
Lineage assignments on the IgG+ memory population of eight healthy donors. (**A**) Separately for each donor, increasing numbers of error-corrected sequences were selected, lineages were assigned, and the frequency of sequences belonging to a lineage with at least two members was calculated. The clonal sequence frequency was plotted, with the dark line indicating the mean clonality of all eight donors, and increasingly transparent bands indicating 1 or 2 standard deviations. (**B**) Using all error-corrected sequences from each donor, lineages were assigned and the size of each lineage was calculated. Lineage size frequencies were then plotted, with the dark line indicating the mean for all eight donors, and the transparent bands representing 1 or 2 standard deviations (in this plot, it is virtually impossible to identify the band that represents 1 SD). Variable (**C**) and diversity (**D**) gene family and joining (**E**) gene use were determined for all sequences (counting each sequence once) and for all lineages (counting each lineage only once, regardless of size). Plots represent the mean ±SEM for each of the eight donors. No significant differences in gene use between sequences and lineages were observed (ANOVA). Lineages were binned by average nucleotide mutation count (**F**), average amino acid mutation count (**G**) and HCDR3 length (**H**), counting each lineage only once. Histograms displaying the lineage frequency for each of the above characteristics were plotted, with lineages from each donor represented as a single, semi-transparent plot. Lineage size was then plotted against three genetic features: average nucleotide mutation count (**I**), average amino acid mutation count (**J**) and HCDR3 length (**K**). No statistically significant correlation was observed between lineage size and any of the three genetic features that were tested (ANOVA).

The combination of error correction and unseeded lineage assignment allows us to broadly characterize clonal lineages in human IgG+ memory B cell repertoires for the first time. We first analyzed the overall level of clonality in the IgG+ memory population for the set of 8 donors. From each donor, increasing numbers of sequences were randomly selected, clonal lineages were assigned, and the frequency of sequences belonging to lineages with at least two members was determined (Fig. 5A). Following a rapid increase in the frequency of clonally related sequences, clonality reaches a plateau at approximately 75%, suggesting that the IgG memory population consists of a relatively small number of frequently occurring lineages combined with a much larger number of less common lineages. To verify this, we determined the distribution of lineage sizes for lineages containing at least two members (Fig. 5B). Across all lineages with at least two members, nearly 50% contain more than 10 members. When considering all lineages, ‘singleton’ lineages containing only a single member comprise 84.5% of the lineage pool.

Several prominent pathogens, including influenza virus and RSV, have been shown to elicit antibody responses with biased germline gene use^32^,^33^,^34^,^35^. Since the majority of the population has been repeatedly exposed to such pathogens, we attempted to isolate the effect of large clonal lineages on the composition of the IgG+ memory repertoire. We first compared the germline composition of lineages to the germline composition of individual sequences. To calculate the germline composition of lineages, we determined the germline gene family used by each lineage, counting each lineage only once regardless of size. In this way, we eliminate the influence of lineage size on the use of germline genes to determine the extent to which large lineages are able to bias the total IgG memory repertoire. The frequency of variable, diversity and joining germline gene family use at the lineage level (Fig. 5C–E) is statistically indistinguishable from the frequency of germline gene family use at the individual sequence level. We next sought to determine additional features related to lineage size. We sorted all lineages by size and compared CDR3 length, nucleotide mutation frequency and amino acid mutation frequency to the lineage size (Fig. 5F–K). None of these features correlated with lineage size, indicating that several prominent genetic features are not skewed by the size of the lineage.

## Discussion

In this report, we describe Clonify, an antibody clonal lineage assignment algorithm capable of operating on large NGS data sets. The Clonify algorithm was benchmarked and the lineage assignments were found to be highly accurate. When applying the Clonify algorithm to error-corrected IgG+ memory B cells, we found a surprising lack of influence of the largest lineages on the total repertoire composition. Together with previous studies^6^,^7^,^8^ showing conservation of high-level repertoire features, the lack of correlation between lineage size and several important antibody genetic features provides mounting genetic evidence for a global control mechanism that regulates overall antibody repertoire composition.

More broadly, the means to perform accurate unseeded lineage assignment will have a profound impact on our ability to study humoral responses to infection and immunization. First and most significantly, it enables the large-scale analysis of antibody clonal lineages as a functional unit of the immune response, as shown in this report. Studying the humoral response at the level of the clonal lineage makes sense both practically and philosophically. In the case of HIV infection, a single or a small number of lineages are typically able to recapitulate serum neutralization activity among donors with broad and potent serum neutralization capacity^23^,^36^. Although lineage members typically display similar functional characteristics, members are often complementary, with lineages displaying broader or more potent activity than any single member of the lineage^37^. Thus, there are substantial advantages to analyses performed on lineages, as opposed to studies with a more narrow focus on individual antibody sequences. Practically, studying the antibody response at the lineage level provides a means for mitigating one of the largest problems in antibody repertoire sequencing: insufficient sampling depth. Typical antibody repertoire sequencing experiments involve obtaining hundreds of thousands or millions of sequencing reads from an aliquot of 5–10 million PBMCs. While this sequencing depth can provide adequate coverage of the antibody genes in such an aliquot, the aliquot itself represents a tiny fraction of the antibody diversity encoded in the circulating B cell repertoire. Attempts at direct comparison between antibody repertoires at the individual sequence level are nearly impossible at such a shallow sampling depth, because observed repertoire differences are overwhelmed by sample variability. Since actively maturing antibody lineages are typically present in sufficient frequency that many members of a single lineage are observed in aliquots of 5–10 million PBMCs, analysis at this level reduces sample-to-sample variability and produces results that are more reproducible and biologically informative.

A further important consideration is that accurate unseeded lineage assignment can be used to provide critical feedback in the design and testing of candidate vaccines. In cases of chronic viral infection such as HIV and HCV, much effort is currently directed toward a greater understanding of the longitudinal co-evolution of virus and antibody and knowledge gleaned from such experiments is expected to provide valuable guidance for rational vaccine design. Although previous work has detailed the longitudinal maturation of individual neutralizing lineages, the effectiveness of such studies can be greatly enhanced by considering the maturation of many anti-viral antibody lineages in parallel, similar to the process described for the healthy donors here. In addition to defining the natural maturation pathway of potentially protective lineages, we must also define the maturation characteristics of lineages that fail to develop broad and potent HIV or HCV neutralizing activity. Inasmuch as the analysis of protective lineages defines a path by which desirable antibodies can be generated, studying the development of lineages with undesirable function enumerates maturation patterns that should be avoided or blocked by a successful vaccine. Complementing the benefit to immunogen design, unseeded lineage assignment is extremely useful when probing antibody responses to immunization. In such experiments, analyzing the total polyclonal response to the vaccinating immunogen is crucial, and can help identify unexpected antibody responses. Especially in the case of rationally designed immunogens, comprehensive analysis of the polyclonal response can highlight immunogenic off-target regions and guide optimization efforts to minimize unwanted responses. Thus, accurate lineage assignment with the Clonify algorithm represents a substantial advance in our ability to study antibody responses to infection and immunization and is a useful tool to aid the rational design and testing of human vaccines.

## Methods

### RNA isolation from IgG+ memory B cells

Peripheral blood was obtained from healthy adult donors following informed consent, under a protocol approved by the Scripps Institutional Review Board. All methods were carried out in accordance with the approved protocol guidelines. Peripheral blood mononuclear cells (PBMCs) were isolated from the whole blood of eight healthy donors by gradient centrifugation (Histopaque-1077; Sigma-Aldrich). From each donor, IgG+ memory B cells were separated from approximately 10 million PBMCs by selective depletion (Switched memory B cell isolation kit; Miltenyi Biotec) and total RNA was extracted (RNeasy; Qiagen).

### Library preparation and sequencing

Approximately 10% of each total RNA sample was subjected to reverse transcription (Superscript III; Life Technologies) using barcoding primers that contain unique antibody identifiers (UAIDs; primer sequences can be found in Supplementary Table 2). The resulting cDNA was purified (Qiaquick; Qiagen) and eluted into 50 uL of water. 10 uL of cDNA was used to amplify antibody heavy chains (HotStarTaq Plus; Qiagen) in a 50 uL total reaction volume using the following thermal cycling program: 94 C for 5 minutes; 30 cycles of 94 C for 30 seconds, 55 C for 30 seconds, 72 C for 2 minutes; 72 C for 7 minutes. Following initial amplification, PCR products were purified using 45 uL of SPRIselect beads (Beckman-Coulter Genomics) per 50 uL PCR reaction and eluted in 50 uL of water. Illumina sequencing adapters and sample-specific indexes were added during a second round of PCR using 1 uL of purified PCR product in 100 uL of total reaction volume and using the following thermal cycling program: 94 C for 5 minutes; 10 cycles of 94 C for 30 seconds, 55 C for 30 seconds, 72 C for 2 minutes; 72 C for 7 minutes. Indexed PCR products were purified using 75 uL of SPRIselect beads and eluted in 50 uL of water. Samples were quantified using fluorometry (Qubit; Life Technologies), pooled at approximately equimolar concentrations and the sample pool was requantified. Samples were loaded onto an Illumina MiSeq sequencer with a target loading concentration of 40pM and 10% PhiX and sequenced (MiSeq 600-base v3 reagent kit; Illumina).

### Initial sequence analysis and UAID processing

Paired-end MiSeq reads were merged with PANDAseq^38^. Germline assignment, junction identification, and other basic antibody information was determined using an in-house antibody sequence analysis pipeline based on IgBLAST^39^. As part of the analysis pipeline, non-functional antibody sequences and other sequencing artifacts were removed and frameshift indels were corrected. Additionally, the initial analysis pipeline was run with the UAID option set to 20 nucleotides (-u 20), which parses UAIDs from each sequence and populates the appropriate field in the JSON output. Output was stored in a MongoDB database for querying and further analysis. The complete initial analysis pipeline, including compiled binaries of IgBLAST for Linux and OSX, is available at www.github.com/bryanbriney/abanalysis. Antibody sequences were then binned by UAID, and all bins containing only a single sequence were discarded. For each bin containing two or more sequences, the appropriate germline variable gene region was added to the bin to serve as a consensus tiebreaker. The bins were then separately aligned with Muscle^40^ and consensus sequences were generated using Biopython (www.biopython.org). Consensus sequences were re-processed with the initial analysis pipeline and stored in a separate MongoDB database.

### Network graphs

Starting with a set of sequences belonging to a single clonal lineage, sequences were organized into nodes with each node representing a unique antibody sequence. Pairwise comparisons were made between each node and all other nodes, and edges were built between each node and the nearest neighbor node. In cases with more than one nearest neighbor, separate edges were constructed for each nearest neighbor node. Node size was adjusted so that node area was proportional to the number of identical sequences represented by the node. Network graphs were plotted in Python using the NetworkX package (https://networkx.github.io/).

### Clonal lineage assignment

Sequences were assigned to clonal lineages using Clonify, a software package specifically developed for antibody lineage assignment. Clonify uses an antibody-specific distance metric, which incorporates length-normalized CDR3 edit distance, variable and joining gene use, and shared somatic mutations to determine the relatedness of each pair of antibody sequences. Clonify was designed to integrate smoothly with our initial analysis pipeline, able to directly query and update a MongoDB database containing pipeline output.

To determine the CDR3 edit distance, Levenshtein edit distance is calculated for each pair of CDR3 sequences, with insertion or deletion (indel) edits penalized twice as heavily as substitution edits, based on the relatively infrequency of indel mutations compared to substitution mutations in the antibody repertoire^41^,^42^. When computing the edit distance for pairs of sequences of the same length, gaps can be introduced which result in incorrectly low distance scores. To compensate for this possibility, Clonify forces gapless distance calculations when comparing CDR3 sequences of the same length. The variable and joining genes are then compared, and a penalty is applied to sequence pairs that encode different variable or joining genes. While clonally related sequences should virtually always encode the same germline variable and joining genes, erroneous germline assignments can occasionally cause clonally related sequences to appear to encode different germline genes. The penalties for different variable gene use (10) and joining gene use (8) were determined based on the estimated frequency of variable and joining gene miss-assignment by IgBLAST^39^,^43^. Each sequence pair is then examined for the presence of shared mutations. For each shared mutation, defined as the same substitution event at the same position, a bonus of 0.35 is applied. This bonus is critical for correct lineage assignment of highly mutated antibody sequences. In highly mutated clonally related sequences, the CDR3 regions may diverge by more than 25%. Clustering such sequences using only CDR3 distance would either miss such related sequences or be so broad as to incorrectly cluster many unrelated sequences. The bonus value was determined based on the estimated ratio of non-CDR3 mutations to CDR3 mutations. Finally, the adjusted pairwise distance score is normalized to the length of the shorter of the two CDR3 sequences.

In order to be able to rapidly process large numbers of sequences, two methods are used to speed sequence processing. First, if a pair of sequences encode different variable gene families, it is extremely unlikely that they are clonally related. Therefore, Clonify screens each pair of sequences and only computes the full distance score if the sequence pair encodes germline genes from the same variable gene family. Second, Clonify has a user-defined option to pre-cluster sequences by variable gene or variable gene family. When using the option to pre-cluster by variable gene, sequences are grouped by variable gene and separate distance matrices are calculated for each group. This allows Clonlify to calculate several smaller distance matrices, which requires far fewer calculations than a single larger distance matrix and can result in dramatically reduced runtime. Clonify is written in Python and the source code is available at www.github.com/briney/clonify-python.

### Graphics

Plots were generated either in Prism (GraphPad) or in Python using the Matplotlib (matplotlib.org), Bokeh (bokeh.pydata.org) and Seaborn (stanford.edu/~mwaskom/software/seaborn/) graphing packages.

## Additional Information

**How to cite this article**: Briney, B. *et al*. Clonify: unseeded antibody lineage assignment from next-generation sequencing data. *Sci. Rep.* **6**, 23901; doi: 10.1038/srep23901 (2016).

## Supplementary Material

Supplementary Information
