# Supplementary material for: Clonify: unseeded antibody lineage assignment from next-generation sequencing data
Source: Sci Rep. 2016 Apr 22;6:23901. doi: 10.1038/srep23901 (PMC4840318; doi:10.1038/srep23901)
Supplement: Supplementary Information [file srep23901-s1.pdf]

SUPPLEMENTARY INFORMATION

Clonify: unseeded antibody lineage assignment from next-generation sequencing data

Bryan Briney[1,2,3*], Khoa Le[1,2,3], Jiang Zhu[1,2,3], Dennis R. Burton[1,2,3,4*]

[1]Department of Immunology and Microbial Science, The Scripps Research Institute, La Jolla, CA 92037, USA. [2]International AIDS Vaccine Initiative Neutralizing Antibody Center, The Scripps Research Institute, La Jolla, CA 92037, USA. [3]Center for HIV/AIDS Vaccine Immunology and Immunogen Discovery, The Scripps Research Institute, La Jolla, CA 92037, USA. [4]Ragon Institute of Massachusetts General Hospital, Massachusetts Institute of Technology, and Harvard University, Boston, MA 02142, USA.

Supplementary Methods

Description of previously published unseeded lineage assignment algorithms (quoting directly from the appropriate publication):

Quake, 2013a: "Protein sequences with the same V and J assignment and with CDR3 region differed by no more than one amino acid were grouped together into a lineage."

Quake, 2013b: "A lineage is formed and populated with one IGH sequence (seed). Then, all IGH sequences in the lineages (initially only the seed) are compared with all other IGH sequences of the same length using the same V and J segments. If their junctional regions (untemplated nucleotides and D segments) are at least 90% identical, the IGH sequence is added to the lineage. This process is repeated until the lineage does not grow."

Boyd, 2014: "Arrangements were assigned to clonal lineages...by clustering, on CDR3 nucleotide similarity, of IGHs that utilized the same IGHV and IGHJ segments and that had the same CDR3 lengths...A rearrangement was assigned to a [clonal lineage] cluster when it was within a Hamming distance equivalent to 95% identity to any sequence in the cluster and where all sequences in a cluster showed at least 80% identity."

Church, 2014: "For most of our work, we chose to use single-linkage agglomerative hierarchical clustering with Levenshtein edit distance as the metric. To make the clustering process more tractable, we partitioned our reads based on VJ identity. Within each partition we then performed sequence clustering using only the CDR3 junction nucleotide sequence. To account for sequencing errors, we examined the cophenetic distances observed in the linkage tree, and determined the optimal distance to clip the trees at 4-5 edits."

Martinez-Barnetche, 2015: "For that reason, we define that 2 reads belong to the same clonotype if the following is true: (1) The V and J gene assignment is the same in both sequences; (2) Junction regions of the 2 sequences have a nucleotide identity >= 97%; ... and (3) The trimmed length of the shorter Junction sequence >= 97% of the length of the larger Junction sequence"

Supplementary Figure 1. Overview schematic of cDNA barcoding. Antibody mRNA is reverse transcribed into cDNA using a gene-specific primer with an overhang that includes the UAID (light blue) and an amplification tag (dark blue). cDNA is amplified with gene-specific primers that anneal to antibody leader sequences with an overhang that contains an Illumina sequencing adapter (green) and a reverse primer that anneals to the amplification tag and contains the other Illumina adapter as an overhang.

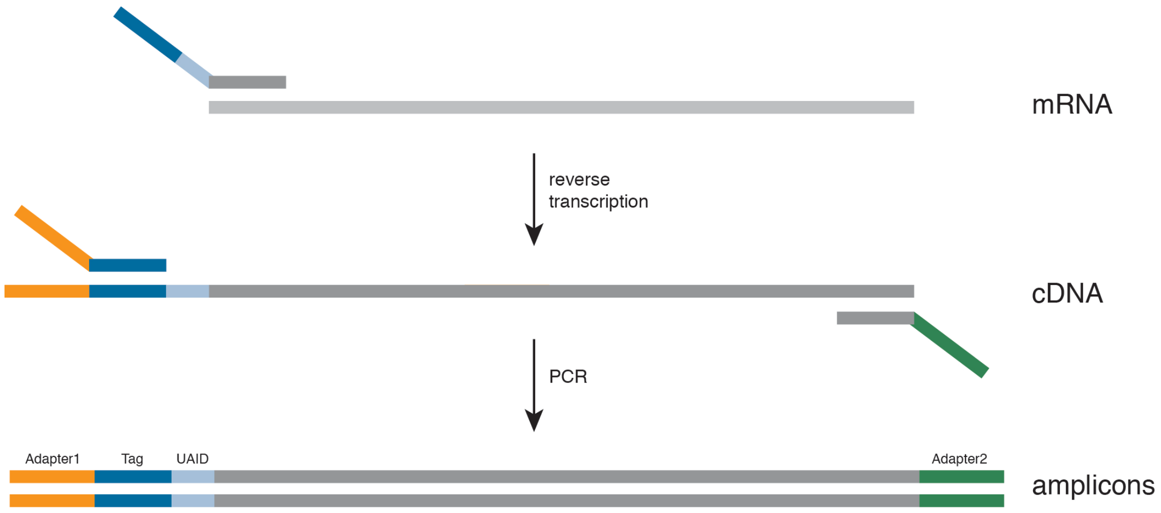

Supplementary Figure 2. Sequence identity of PGT151 family HCDR3s. Amino acid HCDR3 sequences from each PGT151 family member were compared to all other PGT151 family members and the percent identity was calculated. PGT153 has a significantly lower identity to other PGT151 family members.

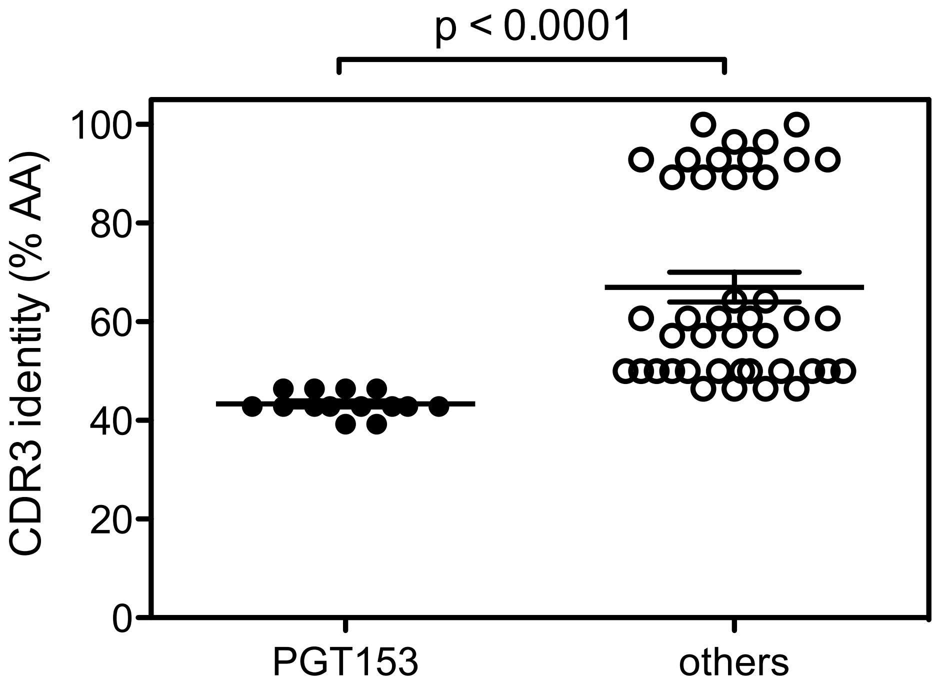

Supplementary Table 1. Per-donor NGS sequencing information.

Donor	Raw Sequences	Error-corrected Sequences
NBD4681	427,086	23,892
NBD4967	655,708	37,802
NBD5146	319,104	24,250
NBD5273	960,251	47,043
NBD5403	188,804	9,884
NBD5492	743,136	37,984
NBD5499	864,424	44,874
NBDQ9P	462,114	26,670

Supplementary Table 2. Primers for UAID barcoding and amplification.

Reverse transcription

IgG: ACACTCTTTCCCTACACGACGCTCTTCCGATCTNNNNNNNNNNNNNNNNNNNNSGATGGGCCCTTGGTGGARGC
IgM: ACACTCTTTCCCTACACGACGCTCTTCCGATCTNNNNNNNNNNNNNNNNNNNNGGTTGGGGCGGATGCACTCC

Round 1 PCR

VH1: AGACGTGTGCTCTTCCGATCTAGCAGCCACAGGTGCCCACTCC
VH2: AGACGTGTGCTCTTCCGATCTAYCCCTTCMTGGGTCTTGTCC
VH3: AGACGTGTGCTCTTCCGATCTTMTTTTARRAGGTGTCCAGTGT
VH4: AGACGTGTGCTCTTCCGATCTGCTCCCAGATGGGTCCTGYCC
VH5: AGACGTGTGCTCTTCCGATCTGTTCTCCAAGGAGTCTGTYCC
VH6: AGACGTGTGCTCTTCCGATCTCTCCCATGGGGTGTCCTGTCA
VH7: AGACGTGTGCTCTTCCGATCTGCAGCAACAGGTGCCCACTCC
Rev: ACACTCTTTCCCTACACGACG

Indexing PCR

XXXXXXXX corresponds to the appropriate index sequence, taken from Illumina's list of TruSeq indexes.

Fwd: CAAGCAGAAGACGGCATACGAGAGATCGGTCTCGGCATTCCTGCTGAAGATGTGACTGGAGTTCAGACGTGTGCTCTTCCGATC
Rev: AATGATACGGCGACCACCGAGATCTXXXXXXXXACACTCTTTCCCTACACGACG

Supplementary Table 3. Germline genes and HCDR3 for PGT151 lineage members.

Sequence	V_H gene	D_H gene	J_H gene	HCDR3
PGT151	IGHV3-30*04	IGHV3-10*01	IGHJ6*02	CARMFQESGPPRLDRWSGRNYYYYSGMDVW
PGT152	IGHV3-30*04	IGHV3-3*01	IGHJ6*02	CARMFQESGPPRFDSWSGRNYYYYSGMDVW
PGT153	IGHV3-30*13	IGHV5-18*01	IGHJ6*02	CARDRDGYGPPQIQTWSGRYLHLYSGIDAW
PGT154	IGHV3-30*04	IGHV3-3*01	IGHJ6*02	CARNIEEFSVPQFDSWSGRSYYHYFGMDVW
PGT155	IGHV3-30*04	IGHV3-3*01	IGHJ6*02	CAKDGEEHKVPQLHSWSGRNLYHYTGFDVW
PGT156	IGHV3-30*04	IGHV3-3*01	IGHJ6*02	CAKDGEEHKVPQLHSWSGRNLYHYTGFDVW
PGT157	IGHV3-30*04	IGHV3-3*01	IGHJ6*02	CAKDGEEHEVPQLHSWSGRNLYHYTGVDIW
PGT158	IGHV3-30*04	IGHV3-3*01	IGHJ6*02	CAKDGEEHEVPQLHSWSGRNLYHYTGVDVW

Supplementary Table 4. Clonify-assigned lineages from putative PGT141-like sequences.

Clonify-assigned lineages are shown, along with lineage size and a representative junction sequence. The lineage that Clonify has identified as PGT141-like is highlighted in red. For reference, the junction of PGT141 is shown directly below the junction of lineage v0_5 and is highlighted in blue.

Lineage	Size (count)	Size (%)	Junction
v0_1	5	1.8	CLVGSKHRLRDYW
v0_2	4	1.5	CAGGSKHRLQDYILYREW
v0_3	3	1.1	CTRGSKHRLRDYVLYDDYGLINYQEW
v0_4	2	0.7	CVGGSNIDYKTTYSTVSGGIIKNMETTLQVW
v0_5	259	94.5	CTRGSKHRLRDYVLYDDYGLINYQEWNDYLEFLDVW
			CTRGSKHRLRDYVLYDDYGLINYQEWNDYLEFLDVW

Supplementary Table 5. Clonify-assigned lineages from putative PGV04-like sequences.

Clonify-assigned lineages are shown, along with lineage size and a representative junction sequence. The lineage that Clonify has identified as PGV04-like is highlighted in red. Four lineages that contain only a single sequence (v0_2, v0_6, v0_11 and v0_15) have been omitted. For reference, the junction sequence of PGV04 is shown directly below the junction of lineage v0_18 and is highlighted in blue.

Lineage	Size (count)	Size (%)	Junction
v0_1	2	0.05	CAREW
v0_3	2	0.05	CARAGVWFGELLPHWSGVGGGMDVW
v0_4	4	0.09	CARKGVIIPGVQIKSDFSGTDSFQLW
v0_5	2	0.05	CARGAFRRVGLADGFDPW
v0_7	3	0.07	CVRDLGGNEEYW
v0_8	5	0.12	CARQKFEKYTGGQGW
v0_9	2	0.05	CARDGGTGPPRYFLYW
v0_10	4	0.09	CAKQKSDSEGFACDLW
v0_12	4	0.09	CARQFYTGGQGWYFDLW
v0_13	2	0.05	CARQRQKFASRYSGDQGSYFDLW
v0_14	22	0.52	CARQTFKPDFYFADQGWSFNLW
v0_16	11	0.26	CARKKREDGFNLYFDLW
v0_17	195	4.6	CARKTKGDVSGDGRGFFFDLW
v0_18	4002	93.8	CARQKFERGGQGWYFDLW
			CARQKFYTGGQGWYFDLW
